# Stand Height Increment from Two-Epoch Aerial Laser Scanning Data and Inventory Data

**DOI:** 10.3390/s25216606

**Published:** 2025-10-27

**Authors:** Paulina Jaczewska, Aleksandra Sekrecka, Bartosz Czarnecki

**Affiliations:** Department of Imagery Intelligence, Faculty of Civil Engineering and Geodesy, Military University of Technology, 00-908 Warsaw, Poland; aleksandra.sekrecka@wat.edu.pl (A.S.);

**Keywords:** LiDAR, point cloud processing, canopy height model, forest management

## Abstract

The use of LiDAR in estimating tree growth is a current and practical research topic that is important from both an ecological and forest management perspective. The aim of this study was to assess the possibility of applying publicly available LiDAR data to assess the growth of forest stands. This study focused on forests in northern Poland, where pine trees dominate, but deciduous trees such as alders and birches are also partially present. The research used generally available point clouds from airborne LiDAR data from the years 2013 and 2022 with an average density of 4 pts/m^2^ and an accuracy of 0.15–0.25 m. Inventory data were obtained for the same dates. A methodology was developed to determine height increments from these data, and 216 corresponding tree stands were compared. The Pearson correlation coefficient was 0.6, showing a moderate correlation between height increments determined from LiDAR and inventory data. Performing LiDAR measurements during the growing season could minimize errors in determining stand heights and increase the correction between airborne laser scanning data and inventory data. Our experiment confirms that it is possible to improve forest inventory and forest management using airborne LiDAR data.

## 1. Introduction

Forests play a key role in achieving the goals of sustainable development. Responsible forest management may contribute to counteracting climate changes, protecting biodiversity, and improving the quality of human life. The protection and sustainable exploitation of forests are the main elements in international agreements, such as the Paris Treaty, with respect to climate change [1]. One of the key elements of forest management is the assessment of the growth of forest stands.

Assessing forest growth enables us to estimate the carbon dioxide content in trees and soil [2]; estimate wood biomass and plan for sustainable forest management [3]; manage timber harvesting and maintain long-term forest health [4]. In addition, knowledge of forest growth allows us to assess the impact of climate change, diseases, and pests on the condition of forests [5,6]. Data on forest growth may provide a basis for prognosing future changes in the structure, mass, and species composition of the forest [7]. These forecasts are indispensable for creating long-term plans of forest protection and management and for evaluating the impact of economic activities on forests [8]. Forests also play an essential role in soil and water protection. Thanks to their crowns and root systems, trees improve the stability of soil, water retention, and the nitrogen cycle. The assessment of tree growth allows for the evaluation how well forests perform these functions, which is important for preventing soil erosion or improving the quality of ground waters [9]. Other applications of the assessment of tree growth include monitoring the influence of human activity, including deforestation, urbanisation, agriculture, or climate change, on forest ecosystems. It enables us to determine how various forms of human activity influence the growth of trees and the overall condition of forests, which may provide a basis for taking protective actions or introducing certain regulations. These analyses provide valuable information about the functioning of forests, their response to environmental changes, interactions between various species, and environmental processes. The results of these studies are used in the scientific analysis of forest ecosystems and may constitute a basis for developing environmental protection policies [10,11,12].

One of the methods for assessing the growth of tree stands is LiDAR (Light Detection and Ranging) technology, which enables non-invasive, highly accurate measurement of the growth of forest stands in large areas, including those that are difficult to access. The main advantages of LiDAR data over photogrammetric data are their independence from the weather, apart from fog and thick cloud cover, and the short data processing time. LiDAR data may be obtained at satellite, aviation, or low altitudes, as well as from ground level. Obtaining LiDAR data from aviation altitude allows for the determination of the main factors of forest stands: tree height (H), crown diameter, crown surface, and volume [13,14,15]. Thanks to the possibility of obtaining detailed spatial data in large areas, LiDAR-based methods enable a precise analysis of the increase in height, volume, spatial structure, and other properties of the forest stand in various periods. This technology is becoming invaluable in modern forest management and monitoring the condition of forests. Tree metrics effectively capture the arrangement of many forest structures, such as crown heterogeneity, the multi-layered profile of the crown, and the openness of the crown. LiDAR data are often used for analysing old-growth forests that are of vital importance for maintaining biodiversity [16]. Another advantage of LiDAR data is the possibility to classify points in the cloud, e.g., to divide them into points representing trees, soil, plant cover, etc. This enables us to precisely determine the changes in forest structure that result from growth. This kind of approach allows for a detailed analysis of growth with respect to individual species [17]. To improve the accuracy of classification of tree species, Hu and Tan proposed the Multiwavelength Airborne Polarimetric LiDAR (MAPL) system, which applies four different LiDAR channels at dual wavelengths and dual polarization—near-infrared (NIR) co-polarization, NIR cross-polarization, green (GN) co-polarization, and GN cross-polarization—to identify and improve the accuracy of tree measurement. Based on the peak intensity values from the MAPL decision tree, an approach was developed to classify the different species of trees [18].

Jin et al. [19] used terrestrial laser scanning to estimate tree growth at different tree levels. Trouvé et al. [20] used airborne LiDAR data to map forest growth stages in the Central Highlands of Victoria (Australia). They emphasized the high quality of LiDAR data, noting that any errors resulted from the old growth definition. Leitold et al. [21] used multitemporal LiDAR data to study forest damage and growth rates after Hurricane Maria in Puerto Rico. They generated a CHM (Canopy Height Model) with a resolution of 1 m and determined height increments at 1 m intervals. In other studies, researchers used ALS (Aerial Laser Scanning) to examine the occurrence of gaps in tropical forests and the growth rate of trees in these gaps. The trees inside the gaps grew 1.5 m per year, while the trees outside the gaps grew more slowly (0.7 m per year) [22]. In turn, Bour et al. [23] combined airborne LiDAR data and time-since-harvest maps to model height growth of post-logged forests. They developed a random forest model in which the forest height is a function of the age of the stand and environmental variables. Their model achieved an RMSE of 19% when comparing predicted and observed heights. Tree height growth using ALS was also studied by Tymińska-Czabańska et al. [24] in terms of determining the mortality factors of Scots pine stands in Poland. On the other hand, the disadvantage of airborne LiDAR data are the high costs of data acquisition, which render this method of collecting data completely unviable for small areas. In light of this, LiDAR data are increasingly frequently obtained from UAV platforms [25,26,27,28]. UAV-LiDAR offers higher density and resolution of the point cloud than airborne LiDAR, which translates into a higher level of detail of the created maps. It also has a higher spatial range than ground LiDAR, higher accuracy than satellite LiDAR, and carries lower personnel risk and costs than airborne LiDAR. Data from UAVs, supplemented by climate, soil, topographic, and radar data, may constitute a perfect source of data for developing large-area Forest Stand Mean Height Mapping [29].

However, a disadvantage of this solution is the fact that, as in the case of airborne LiDAR, the laser beam from the scanner installed on the UAV platform is unable to reach the undergrowth plants. This makes it significantly more difficult (yet not impossible, as was proven in [21]) to determine the diameter at breast height (DBH). Zhang et al. [30] used ALS to develop a CHM, and they also assessed the correlation between ALS and ULS (Unmanned Laser Scanning) and the possibility of their mutual integration. Komárek et al. [31] concluded that the LIDAR data from UAVs are sufficient for FCH modelling, and that the ALS data do not make significant changes. However, it should be noted that UAV data are collected for small areas. For large areas, the use of UAVs would be too time-consuming and costly. Therefore, in our research, we focused on checking whether LiDAR data alone can be used to assess the height growth of forest stands.

As opposed to UAV LiDAR, terrestrial LiDAR can acquire the dense and high-precision point clouds of trees at ground level, but its spatial coverage is extremely limited [32,33,34,35,36]. Mobile TLS uses highly precise Simultaneous Localization and Mapping (SLAM) algorithms and inertial navigation systems to enable the merging and mapping of data in real time. Although Mobile TLS improves operational capacity, the scanning range is limited to 20 m, and the occlusions of trees often lead to missing data [37,38,39]. Therefore, in order to obtain comprehensive information about forest plants, it is recommended to integrate data from airborne and terrestrial sources. Fusion of UAV and backpack LiDAR is used increasingly often to conduct detailed measurements [40,41,42]. Certain solutions exist that combine the advantages of UAV LiDAR and LiDAR SLAM. They consist of integrating the clouds after they have been transformed to voxel form [38]. Thanks to the high accuracy and efficiency of measurement, the assessment of the growth of forest stands with the use of LiDAR data is becoming increasingly popular in forestry.

The purpose of this study is to analyse the consistency of inventory data and ALS data in terms of assessing forest stand height increments.

In our experiment, we pose the following research questions:

1. Can the increment in forest stand height be determined on the basis of ALS and are the results similar to those calculated on the basis of inventory data?
2. Can ALS data replace inventory data and streamline the inventory process in forests?

This article is structured as follows. Section 2 contains the characteristics of the test area, the measurement LiDAR data used, and the reference data from the state forest database. This section also describes the method used to process the above-mentioned data and method applied to determine the height increment of forest stands. Section 3 presents a detailed description of the obtained results. Section 4 contains a discussion of the obtained results and references to similar research projects. Finally, Section 5 contains the summary and conclusions.

## 2. Materials and Methods

In this study, we focus on comparing the height increments of the forest stands determined using ALS with the corresponding increments calculated from inventory data collected in forest documentation. Height increments were determined between current (2022) and archived (2013) data for the Zbrzyca study area in northern Poland (Section 2.1). In Section 2.2, we describe the data used and the methodology for processing them. The methodology for assessing the increase in tree height consists of two main stages. First, data from forest documentation should be appropriately prepared and historic data should be adjusted to match the structure of current data. Secondly, LiDAR data must be processed to enable us to read the height of the selected forest stand.

### 2.1. Research Area

**Figure 1:**
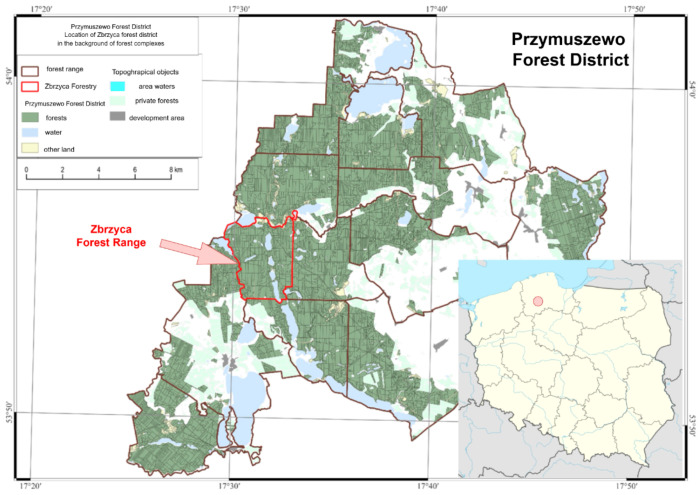
The location of the Przymuszewo Forest District in Poland, with the forest complexes of the Przymuszewo Forest District.

In Poland, the organisational structure of the State Forests distinguishes 429 forest districts, which are further divided into forest ranges, divisions, sub-divisions, and forest units. The test site was the forest range Zbrzyca in the Przymuszewo Forest District, which is located in the northern part of Poland (Figure 1). It is a territorially consistent, uniform forest complex that contains only forests managed by the State Forests, without forests owned by other entities. The forest range covers a surface of 1336.55 ha, of which 1232.47 ha is managed by the Przymuszewo Forest District. They include the following:

- 1162.93 ha of forest areas, areas related to forest management, and wooded and shrubbed areas;
- 24.04 ha of arable land;
- 15.52 ha of land under water;
- 1.33 ha miscellaneous land;
- 0.05 ha developed land;
- 28.60 ha of idle land (including marshes that are protected as conservation areas).

**Figure 2:**
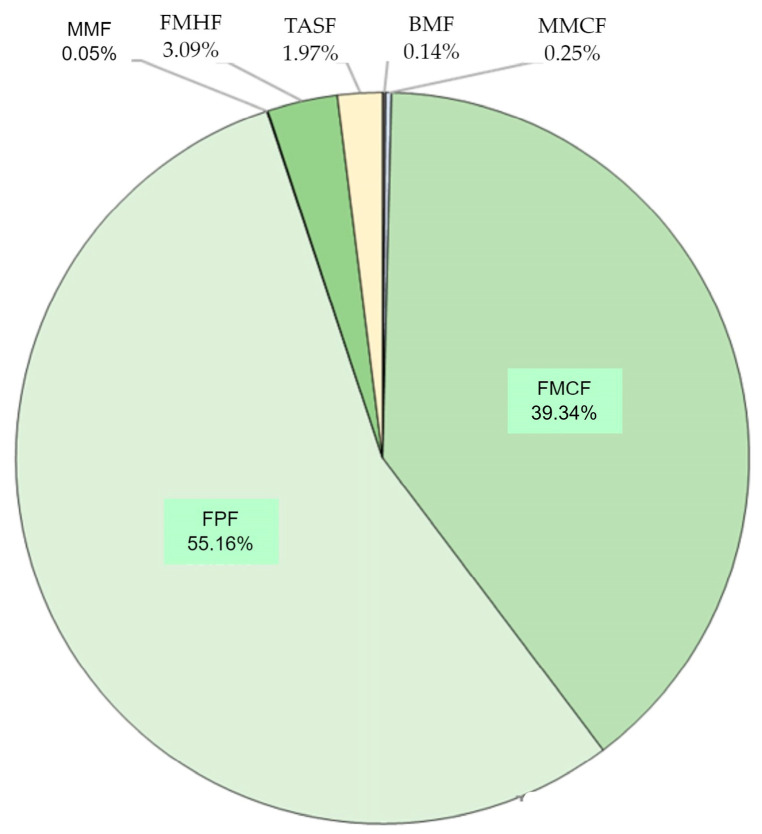
Forest site types of the forest in the Zbrzyca forest range (based on the data from SFIS, Poland).

According to the latest data in the State Forest Information System (SFIS), in terms of surface area, in 96.7% of the forest stands in the Zbrzyca forest range, the dominant species is the European Scots pine (*Pinus sylvestris)*, while in 1.7% it is alder (*Alnus glutinosa*), in 0.7% it is birch (*Betula pendula)*, in 0.5% it is oak (*Quercus)*, in 0.3% it is spruce (*Picea*), and in 0.1% it is larch (*Larix*). Beech (*Fagus*) is not the dominant species in the forest stand of the Zbrzyca forest range, while it is present in other forest ranges—0.7% for the whole forest district. Regarding wood capacity, the share of pine in the forest stands of the Zbrzyca forest range is 94.9%. Such a high share of the European Scots pine is connected to the structure of forest habitats in the test area, which is dominated by fresh coniferous forest and mixed fresh coniferous forest. Figure 2 shows the percentage share of habitat types in the study area. Fresh pine forest (FPF) (55.16% of the total study area) and mixed fresh mixed coniferous forest (FMCF) (39.34%) dominate. The remaining part (5.50%) consists of mixed moist forest (MMF), fresh mixed hardwood forest (FMHF), typical alder swamp forest (TASF), boggy mixed forest (BMF), and moist mixed coniferous forest (MMCF). Of the above habitat types, only TASF has a dominant species other than pine, and it is alder.

The presented structure of habitats is related to the soil conditions in the area. The majority of the forest area is situated on outwash and fluvio-glacial formations. These are mainly podzolic and rusty soils that were formed on gravel and sand.

### 2.2. Data and Processing Methods

In this study, we used publicly available archival and current airborne LIDAR data to assess the growth of forest stands. In our experiment, we verified their consistency with inventory data that were obtained from the resources of forestry documentation. Information on the height of forest stands, the forest unit layer, and the layer of non-exclusive areas (NEAs) was required. For the year 2022, the layers of forest unit and forest stand height were collected from the Data for Reports on Forest Condition (DRFC) integrated with the SFIS ()State Forest Information System. Data for the year 2013 were obtained from forest documentation archives maintained by the SFIS. A detailed description of the forest documentation data is provided in Section 2.2.1. Section 2.2.2 describes LIDAR data and the methodology for assessing height growth based on these data.

#### 2.2.1. Inventory Data from Forest Documentation

Inventory data are obtained during periodic field measurements carried out in forest districts as part of the forest management plan review and updated annually. During the inventory, the condition of forest stands is updated. One of the parameters updated is the height of forest stands. Inventory heights are determined on the basis of field measurements, usually taken with a hypsometer. According to the Forest Management Manual in Poland [43], the average height is determined on the basis of measurements of approximately 10 representative trees, separately for each species in the stand. The height value is rounded to 1 m. Inventory data are evidenced in the State Forest Information System (SFIS).

Since 2010, all the forests managed by the State Forests of Poland have had their geospatial representation recorded in the form of a Forest Digital Map (FDM). An FDM contains a spatial representation of all objects in the forest district in a coordinate system, linked to a database. Each forest district is obliged to update the map annually in terms of economic events and ownership title. The updates are recorded in the State Forest Information System (SFIS). The data, updated annually by forest districts (collected in FDM and SFIS), are published by the Data for Reports on Forest Condition (DRFC) and are available for downloading in form of the ESRI shapefile files, WMS and WFS services, and attached to the national geoportal. The only data made available are layers of ranges of forest districts, forest ranges, forest divisions, and forest units. Vector layers are accompanied by a table data package which includes identification data of the forest unit, natural and habitat data (such as habitat type, tree stand type, forms of nature protection), and stand assessment data (such as species composition, age of main species, site classification, stand density, stand height, growth). The WFS service only publishes current data. Historic data (however not older than before 2015) may be downloaded only from the DRFC website.

This study is based on two out of many thematic layers that are available in the FDM, whose scope of data is related to the analysed issue: the layer of forest units and the layer of non-exclusive areas (NEAs). Forest units are the basic unit of area division of land under the administration of a forest district, defined in terms of their boundaries and assigned a unique forestry address in the country. For forested forest areas, they are separated based on the occurrence of differences in certain properties of forest stands, including the dominant species in the forest stand and the need to establish appropriate economic guidelines for a given part of the forest stand, ensuring the appropriate accuracy of taking inventory of wood resources [44,45,46].

Adjacent forest compartments may have the same dominant species, but may differ in age, vertical structure, ways of forming the forest stand, the origin of the forest stand, the share of co-dominant and admixture species, the dominant type of density, the class of damage to the forest stand, forest site type of the forest, and the site quality class of the forest stand, understood as a difference in height up to 5 m. In general, all of the above factors should be consistent within a single forest unit. For example, pine is the dominant species in a significant part of the study area. However, despite the uniformity of the dominant species, the area is divided into units due to differences in other (above-mentioned) characteristics. If a fragment of forest that stands out from its surroundings is too small to be treated as a separate unit, it is classified as a non-exclusion area (NEA). Non-exclusive areas are small areas occurring in the forest stand whose properties are different than those of the surrounding forest stand, yet they do not meet the criteria to be considered separate forest units. They include gaps or open spaces devoid of trees, renewed and unrenewed sites of a surface area up to 50 acres, created as a result of polycyclic harvesting, and clusters of trees that differ from their surroundings in terms of species or age which qualify as separate forest units but do not meet the surface criterion.

In order to conduct the research presented here, data for the year 2022 (forest unit and forest stand height layers) were collected from the DRFC. The forest unit layer is distributed with tabular data on the properties of species in forest stands (including information about height). Data for 2013 are not available from the DRFC; therefore, the forest unit layer for that year was obtained from the archives of the Przymuszewo Forest District. As the data on the layers do not contain information about the height of the species, tabular data about height were attached (collected from the SFIS database in the form of text files). The non-exclusive area (NEA) layer for both periods was obtained from the archives of the forest district, as data on this layer are not distributed by the DRFC. This metric was used to filter out the emerging height points of tree crowns situated outside the main area. This allowed us to eliminate the influence of trees that do not belong to the core of the forest stand on its height.

**Figure 3:**
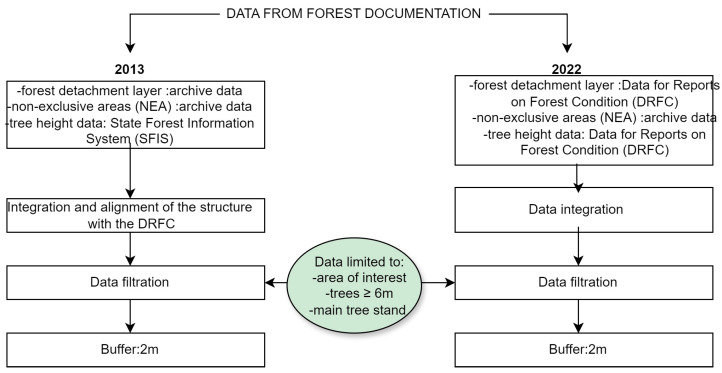
The process of preparing data from forestry documentation (historic data—data from the archives of the Forest District, SFIS—State Forest Information System, DRFC—Data for Reports on Forest Condition based on SFIS).

Analysed years belong to different revisions of the forest management plan and have different sources of data (DRFC or archive data of forest district), which is why there are discrepancies in the data structure. We took this discrepancy into account in our experiment. In the first stage of preparing the inventory data, we adjusted the data to have the same structure so that further work for both dates could proceed in the same way (data integration, filtration, and buffering). The process of preparing forestry data is presented in Figure 3.

Firstly, all the necessary data from both years had to be merged. The layers of forest units and the NEAs are necessary for the analysis. As such, geometric objects that define the borders between forest stands that differ in terms of survey properties should be accompanied by information about the height of the forest stand. For newer data (from 2022), the information about height is integrated with the borders of forest units and published by the DRFC. However, as historic data require the appropriate data structure, the vector data (forest unit layer) were accompanied by a table with the heights of forest stands. Moreover, the attributes of the forest unit layer from the year 2013 are different from those of the files currently published by the DRFC. In order to maintain consistency with newer data, an additional table was attached, containing the description of the dominant species of the forest unit, type of surface, surface area, forest site type, etc. A table with forest addresses was also added to the forest unit layer to enable unambiguous identification of the location of forest units, which, in turn, allowed us to compare the increases in height based on forestry data and LiDAR data.

Analysis of large amounts of data requires setting certain objectives that will enable us to reduce the resulting amount of data, i.e., to remove data that are redundant from the point of view of the subject of the research. First of all, all layers with their attributes were limited to the area of interest (the Zbrzyca forest range). Apart from that, it was necessary to narrow the scope of data in the research area. Excess data (such as the name of the forest stand layer, the number of species in the order of presence, the thickness of large timber in the forest unit) were removed. These other parameters of tree stands are important in forestry and appear in the attribute table of the forest unit layer, but they are not useful for us in this study (in this experiment, we focus only on the analysis of height increments in the forest units). The timber volume and forest stand growth tables [47] used in forestry show the thickness of large timber (i.e., the volume of the tree stand and its increase) starting from the age of 20 years and a height of 6 m. As such, for the purposes of this study, points (trees) lower than or equal to 6 m were rejected [8].

Excess attributes that were irrelevant for the conducted analysis were removed from the forest unit and NEA layers. An index of the year of origin was added to the other attributes in order to distinguish between them in further analyses. Data were filtered to remove layers other than the forest stand and the floor, so as to leave only the main height of the tallest layer of the forest. Then, all species apart from the dominant species were filtered out. The final stage involved creating a buffer around the NEA layers. Due to the origin of this layer (GNSS measurements taken by field workers of the SF with tourist class receivers), significant discrepancies between the position, size, and shape of the objects and the actual objects may be observed. In order to eliminate error, layer objects were enlarged by a 2 m wide buffer (half of the width of the average crown of a mature European Scots pine). Data preparation resulted in creating historic and current vector layers of forest units and non-exclusive areas with the same data structure. These layers only contained the area of interest and the data that were essential for further analysis. Both resulting forest unit layers contained information about the height of forest stands.

In order to calculate the increase in height, it was necessary to merge the data from the analysed years. Due to the changes in the spatial division (resulting from different revisions of the forest management plan) that consisted of dividing forest units and the resulting changes in the addresses of forest units, the data had to be merged from a spatial perspective (not based on attributes, according to the forest address). Pursuant to the principles of creating forest units, they may be merged, provided that the difference in age does not exceed the values specified for the given range. As a result, in 2022, some of the forest units that were separated from neighbouring areas in 2013 constituted single forest units, with the age averaged for the whole forest stand. The borders between forest units also changed between the two revisions as a result of economic events (logging) or due to other reasons (separating conservation areas, e.g., nest protection areas, adjustments in the geometry of land plots in the land register). Considering the above, 236 forest units were selected (out of 458 in 2022 and 451 in 2013) based on the analysis of the product of forest units from the years 2013 and 2022. For these forest units, the increase in height based on SFIS data and those calculated from LiDAR data were then compared. Later, the multiplication of polygon layers with attached data on the height and number of trees from the years 2013 and 2022 was performed. Due to the geometric differences in the contours of objects, the emerging layer contained over five times more objects than the input layers, many of which had a negligible surface area. In order to distinguish the objects that contained the main area of overlap of forest units from the analysed years, spatial selection of the intersection with the scoring layer of the poles of inaccessibility of the selected separations was performed.

**Figure 4:**
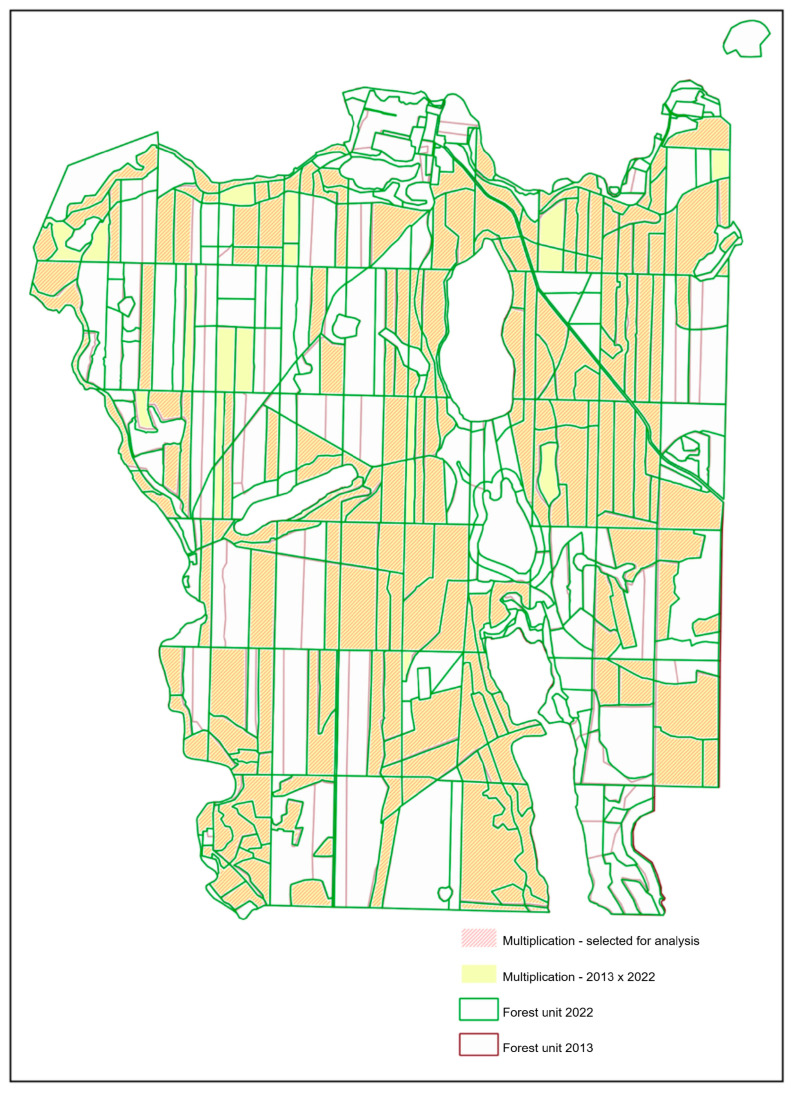
Forest units selected for comparison.

Data analysis consisted of comparing the ages of forest stands. The boundaries of the cuttings did not significantly change their shape. However, between the performed LiDAR scans (9 years), the forest stand may have been cut, as can be seen by the age attribute of the main species. This may be noticed as a result of using the attribute of age of the dominant species. Forest units that had a negative age difference between 2022 and 2013 were removed from the selected data. Forest units that were devoid of trees in 2013 (cuts) were also rejected. Therefore, 218 forest units remained to be analysed. The analysed forest units did not include those that had been classified as forest units with miscalculated height in the previous analyses. The forest units selected for analysis are presented in Figure 4.

#### 2.2.2. ALS Data

LiDAR (Light Detection and Ranging) measurement data from airborne laser scanning (ALS) are a representation of the terrain in the form of a cloud of measurement points of the specific coordinates XYZ. The files are saved in LAS format, and apart from point coordinates, they contain, among others, information about the class of a given point and the intensity of signal reflection. Points may also be assigned RGB values (corresponding to red, green, and blue) obtained from aerial photos. The LAS file format, developed by the ASPRS (American Society for Photogrammetry and Remote Sensing), has become the industry standard for the storage of data from airborne laser scanning.

**Figure 5:**
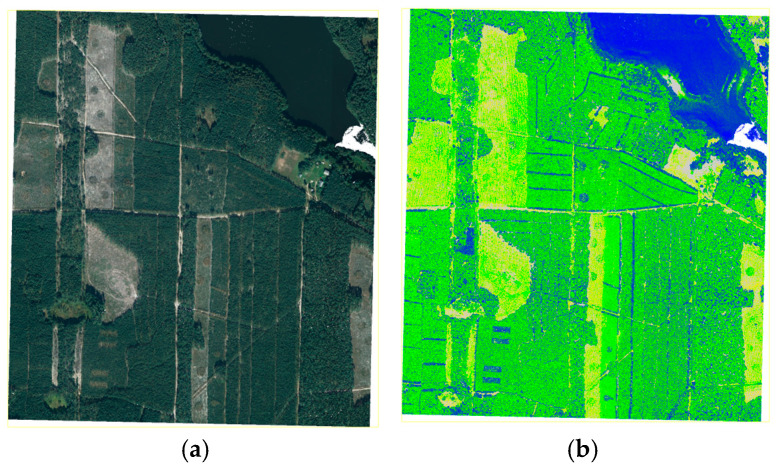
LiDAR data in (**a**) natural colours (RGB) and (**b**) return intensity.

The research was conducted based on LiDAR point clouds with an average density of 4 pts/m^2^ and an accuracy of 0.15–0.25 m. Each point in the LiDAR data cloud saved in LAS format has the following attributes: coordinates (X, Y, Z), intensity, Number of Returns, Classification, Scan Angle Rank, GPS Time, and RGB/RGB + NIR data (optional). Figure 5 presents the point cloud from airborne laser scanning in natural colours and coloured according to increasing return intensity. The information on return intensity is particularly useful for the classification of points in the cloud.

**Figure 6:**
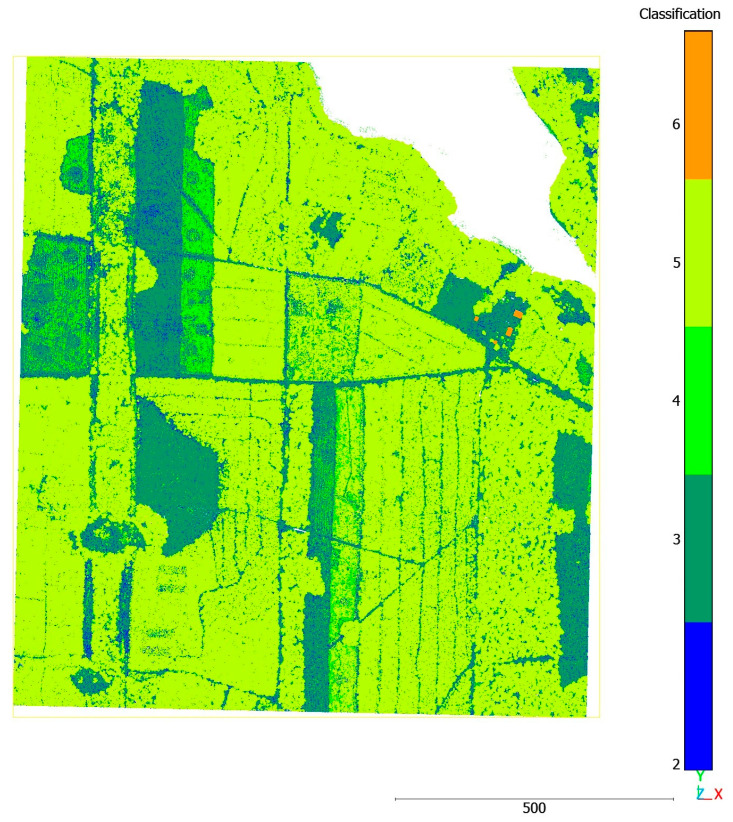
LiDAR data according to distinguished classes: 2—points situated on the ground, 3—points representing low vegetation, 4—points representing medium vegetation, 5—points representing tall vegetation, and 6—points that represent buildings.

In LAS files, classes defined by the ASPRS Standard are distinguished in the form of classification codes. These are numerical codes assigned to a point, specifying that a point belongs to a defined class of objects (e.g., soil, low vegetation, building, or water), as shown in Table 1. Point classes allow us to distinguish various types of objects situated above ground and the ground itself. Figure 6 presents the data from airborne laser scanning for the area of interest, where the subsequent colours represent the corresponding classes, as presented in Table 1.

**Figure 7:**
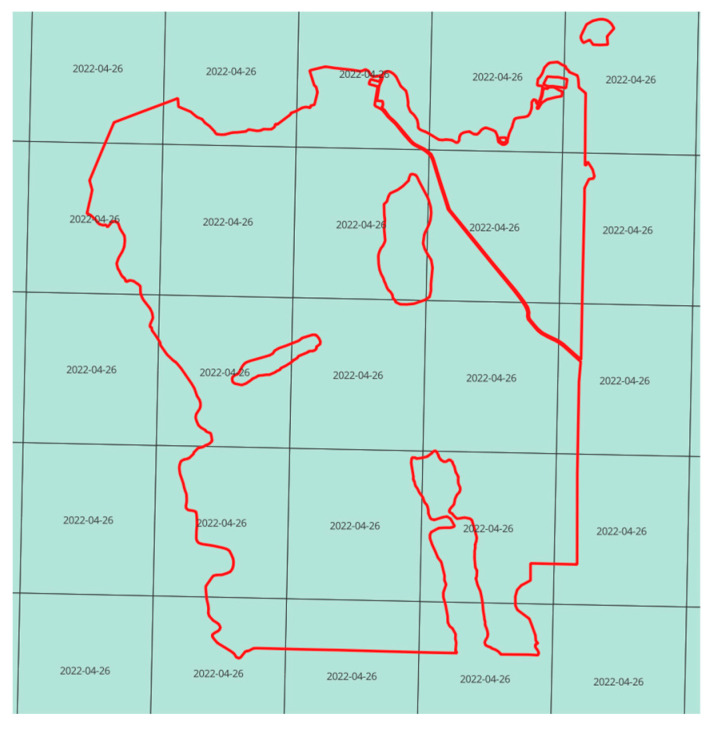
Range of lands owned by State Forests of Poland in Zbrzyca forest range on background of LiDAR data charts.

Historic (7 March 2013) and current (26 April 2022) LiDAR data were recorded in the winter season, i.e., after the end of growth increases from the previous year and before the start of growth in the current year. The data were obtained in the form of several charts of the map that covers the area of the Zbrzyca forest range (Figure 7). Each chart corresponds to a separate LAS file. Later, all files were combined to form consistent sets of historic and current LiDAR data for the whole analysed area.

**Figure 8:**
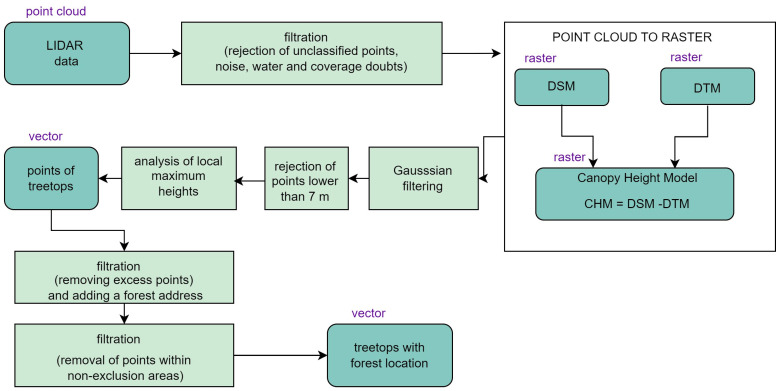
Flow chart of methodology of LiDAR data processing.

LiDAR data may be useful, provided that they have been prepared correctly. The illustration below (Figure 8) presents a flow chart of the methodology of LiDAR data processing in order to determine increases in the height of forest stands. When capturing laser data, apart from the object of interest, reflection from other objects and noise that interfere with the point cloud are recorded. Such interference hinders data analysis, making it less accurate as a result. In order to eliminate such errors, filtration of the point cloud was performed. Unclassified points, noise, water, and coverage doubts were rejected.

The next stage consisted of converting the point cloud to raster form. Two models were developed based on the point cloud: DSM (Digital Surface Model) and DTM (Digital Terrain Model). Both models were calculated with a resolution of 0.5 m, with the use of Delaunay triangulation and linear interpolation during the creation of a grid of triangles from Equations (1) and (2). The DSM was calculated based on the first returns (from tree crowns), while the DTM was based on the farthest returns (from the ground).

(1) $${\;DSM}_{\left(i,j\right)}=TIN\left({H_{first}}_{\left(i,j\right)},r\right)$$

(2) $${\;DTM}_{\left(i,j\right)}=TIN\left({H_{last}}_{\left(i,j\right)},r\right)$$

where ${H_{first}}_{\left(i,j\right)}$ and ${H_{last}}_{\left(i,j\right)}$ is the height of the point in the LiDAR cloud at the point of the coordinates (*i*,*j*), respectively, for the first and last return; *TIN* is the algorithm for the interpolation of the grid of triangles (Delaunay triangulation); *r* is the DSM and DTM spatial resolution. For the purposes of this study, *r* = 0.5 m.

Then, the CHM (Canopy Height Model) was determined from Equation (3). The CHM to is a differential raster of the DSM and the DTM, with a resolution and range identical to those of the models.

(3) $${CHM}_{\left(i,j\right)}={DSM}_{\left(i,j\right)}-{DTM}_{\left(i,j\right)}$$

To minimise the influence of outliers on the model, Gaussian filtration was applied with the smoothening filter, marked as F, based on Equation (4):

(4) $${CHM^{\prime }}_{\left(i,j\right)}=\;\sum_{m=-1}^{1}\sum_{n=-1}^{1}F_{m,n}\;\cdot \;{DECM}_{i+m-1,j+n-1}$$

where ${CHM^{\prime }}_{\left(i,j\right)}$ is the result of Gaussian filtration for point coordinates (i,j), while (m,n) are the coordinates of kernel F (Equation (5)):

(5) $$F=\left[\begin{array}{ccc} 0.0625 & 0.125 & 0.0625\\ 0.125 & 0.25 & 0.125\\ 0.0625 & 0.125 & 0.0625 \end{array}\right]$$

Excess data should be removed from the smoothened Canopy Height Model (CHM), similar to the removal of data from forestry documentation resources. This is why objects (trees) lower than 7 m were cut out of the model and replaced with NA values (Equation (6)):

(6) $${{CHM}^{\prime \prime }}_{\left(i,j\right)}=\;\left\{\begin{array}{cc} NA, & {{CHM}^{\prime \prime }}_{\left(i,j\right)}<7\;m\\ {CHM^{\prime \prime }}_{\left(i,j\right)}, & {CHM^{\prime \prime }}_{\left(i,j\right)}\geq 7\;m \end{array}\right.$$

The last step in LiDAR data processing was the detection of tree tops and exporting them as points to the vector file. In order to detect tree tops, the f(x) function was defined that regulates the size of the area of the raster, in which local maximum heights are sought—interpreted as the tree tops (Equation (7)).

(7) f(x)=0.16x+0.8 

**Figure 9:**
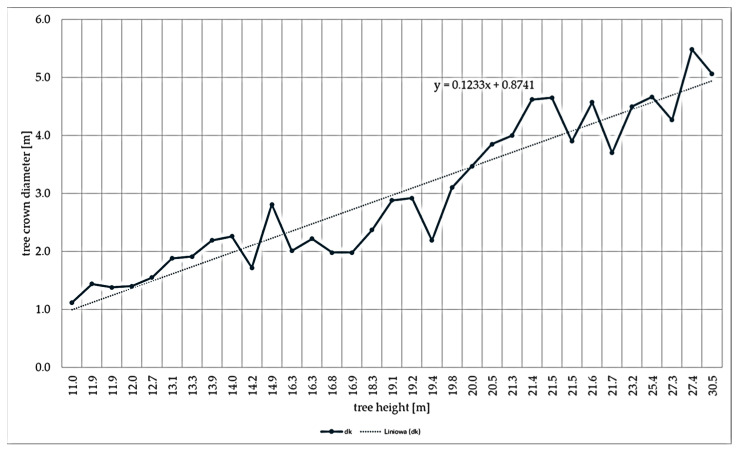
Diagram of relation between crown size and tree height along with trend line.

In order to determine the directional derivative and the free expression of the function, the available data sources on the relation between the height of a tree to crown size were analysed. Figure 9 shows the relationship between crown size and tree height, along with the trend line.

The stages of data processing resulted in vector files in shapefile format that contain the point geometries of the detected tree tops with the attributes of identifier ID, tree top height, and the radius of search for the local maximum height. These vectors contain excess points that are located outside the forests managed by the Przymuszewo Forest District, Zbrzyca forest range. However, they lack an identifier that would locate the given tree top in the forest unit. As such, the tree top vector was filtered to remove irrelevant information. After removing excess points located outside the land of Zbrzyca forest range, points situated in forest units but not forest stands (marshes, woodlots in fields, pastures, and meadows, etc.) were also rejected, as well as points located in non-exclusion areas. Additionally, the forest address was added to identify the precise location of the forest stand and link it to a specific forest unit.

In order to compare the results of LiDAR data processing to the work of a surveyor (who selects several representative trees for measurement, and therefore, in general, rejects trees that are significantly lower or higher than average), a weighted average was calculated, taking into account the number of occurrences of a given height in the specific forest unit. Firstly, the height was rounded up to full meters (according to the accuracy of measurements taken with the use of other methods and to the data that are used in the tables of thickness, volume, and growth). This enabled us to count the number of occurrences of each height in a given forest unit. The weighted average for each forest unit was calculated after the data were merged with the forest unit layer and the forest address key. This enabled us to avoid performing the calculations for several hundred thousand points. The weighted average was calculated from the general formula shown below (8):

(8) $$\bar{h}=\frac{\sum_{i=1}^{n}w_{i}h_{i}}{\sum_{i=1}^{n}w_{i}}$$

where [h1, h2, … hn] is the set of tree heights in the forest unit; [w1, w2, … wn] is the set of weights of the occurrences of a given height in the forest unit that determines the number of occurrences of height $h_{i}$ in forest unit A. The weight $w_{i}\;$is calculated as follows (9):

(9) $$w_{i}=count(A,round(h_{i},0)).$$

$w_{i}$ is the weight corresponding to the number of occurrences of a given height $h_{i}\;$in forest unit A.

This stage results in a vector layer, in which the points indicate individual tree tops. Then, for each forest unit, the weighted average height, which corresponds to the height of the forest stand in the forest unit, was calculated.

## 3. Results

This section presents the results of research on the possibility of applying airborne LiDAR data to assess the increase in the growth of forest stands. Section 3.1 provides a comparison of the average heights in forest units calculated from LiDAR data with inventory heights. This comparison was performed separately for historic data (2013) and current data (2022). This is discussed in the subsequent sub-sections. Section 3.2. presents the analysis of increases in height from 2013 to 2022. In Section 3.3, we describe the analysis of habitat conditions in forest units.

### 3.1. Average Height According to Inventory Data Compared with LiDAR Data

This section presents a comparison of the average heights of forest stands for the same moment in time, separately for historic and current data. The analyses were conducted based on two variants: focusing on the forest site type of the forest and on the dominant species.

#### 3.1.1. Comparison of Average Heights for Historic Data

**Figure 10:**
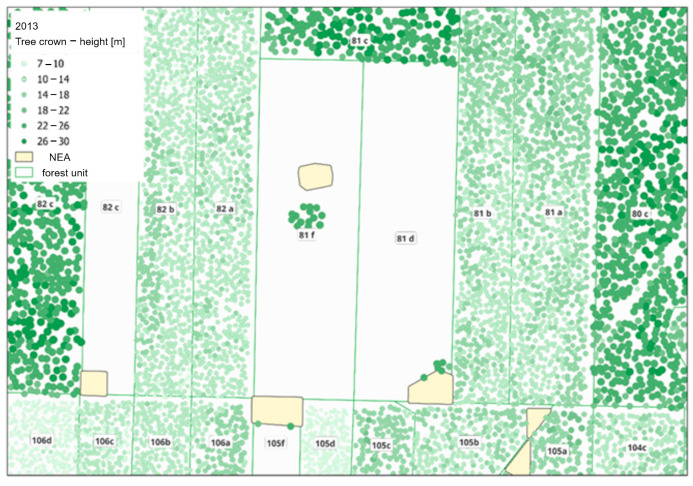
Example of heights estimated incorrectly due to incorrect position of non-exclusion area.

In order to compare the average heights from SFIS and LiDAR data, the height provided in the SFIS was deducted from the average height from LiDAR, rounded up to full meters. For data from 2013, the largest negative differences reached 5 m, and the largest positive ones reached up to 24 m. Positive values that ranged from 8 to 24 m were identified as errors. They occurred in 11 forest units, over a surface area of 19.01 ha, which represents 1% of the analysed area. These errors were identified based on the assessment of the number of identified crowns in a given forest unit, the age of the forest stand according to SFIS data, and the visual analysis of the point layer of tree tops on the background of the layer of contours of forest units and NEAs (Figure 10). The number of crowns identified in forest unit 2 to 59 indicates a density of trees that does not comply with the structure of the forest stand. The age of the forest stand (2–12 years) indicates that the height of trees should not exceed 7 m, i.e., the height of the threshold below which classification was not performed. The analysis of the above factors revealed the following errors:

- Incorrect positioning of the NEA (cluster) on the map; as a result, the height of trees left in the cluster was assumed instead of the height of forest stand;
- Incorrect contours of the forest unit;
- The presence of residual trees, i.e., single trees from an older forest stand, not constituting a cluster (NEA) in the forest unit.

Figure 10 shows the errors in the assessment of tree height. The heights of tree crowns are marked in green. The darker the shade, the higher the trees. In some places (for example forest unit 81 f or 81 d), groups of trees, whose heights are noticeably different from their surroundings, are visible. These are clusters that should belong to the non-exclusive area. Incorrect locations of NEAs do not overlap with these clusters, which leads to errors in determining the height of trees. Further on, this will result in errors in estimating growth. Moreover, for forest units 81 c and 82 c, there is a risk that their contours are incorrect, as the borders of the forest unit are not the same as the borders of a group of trees that are over 26 m high.

**Figure 11:**
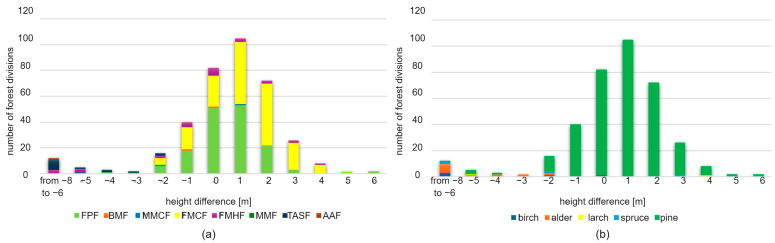
Number of forest units in height difference classes for data from 2013. Forest stand classification based on (**a**) forest site type and (**b**) dominant species.

The differences in crowns that were classified as errors were not taken into consideration in the assessment of differences in the height of forest stands determined based on forestry data and LiDAR. In order to perform this assessment, the number and surface area of forest units in consecutive classes of height differences were compared. Fourteen classes were distinguished, whose names indicate the value of the difference in meters. The first class refers to height differences from −8 m to −6 m, while the subsequent classes designate height differences from −6 to 6 m, with an interval of 1 m. The analysis was conducted taking into account two types of classification of forest stands: the type of forest site and the dominant species. The results are presented in the diagrams below (Figure 11) and in Table 2 and Table 3.

Negative differences mean that the heights determined from laser data were lower than those recorded in the SFIS. A total of 271 forest units with a total area of 852 ha met the required measurement accuracy (difference from −1 m to 1 m), which accounts for 77% of the analysed area. Negative values from −2 m to −5 m were found in 54 forest units (142.11 ha, 13% of the analysed area). Positive values ranging from 2 m to 6 m were noted in 48 forest units (95.72 ha—9% of the analysed area).

In the classification based on types of forest habitats, 19 forest stands with the largest negative error were selected. The errors resulted from the fact that the height of the forest stand from LiDAR data had been underestimated. The errors ranged from −5 m to −3 m. This group mainly includes forest stands situated in fertile and water-related habitats, including the following:

- Fresh mixed hardwood forest (FMHF)—six forest units (accounting for 20% all forest stands of the FMHF type);
- Typical alder swamp forest (TASF)—eight forest units (accounting for 67% all TASF stands);
- Ash alder forest (AAF)—one forest unit (i.e., 100% of all AAF stands);
- Fresh mixed coniferous forest (FMCF)—three forest units (accounting for 0.6% all FMCF stands);
- Fresh pine forest (FPF)—one forest unit (accounting for 2% of all FPF stands).

In the classification based on dominant species, this group (with the largest negative error) included the following forest stands: birch—three forest units, alder—seven forest units, larch—one forest unit, and pine—eight forest units. The numbers represent, respectively, 60%, 58%, 100%, and 2% of all forest units of the same type.

The diagram (Figure 11) reveals that the differences take a Gaussian distribution, where the most frequent value of height difference is 0. The average difference in height for both variants of classification of forest stands is 0.0 m. The standard deviation is 1.9 m for classification based on forest site type, and 0.6 m for classification based on dominant species. This means that no systematic shift was noted, and therefore, the data from forestry documentation and LiDAR data are comparable. A relatively low standard deviation means that the variety of differences in height is low. This, in turn, points to high convergence between the data.

The forest units differ in terms of surface area. In some cases, the surface area of a single forest unit may be larger than the total surface of two other units. As such, the results of the above analyses were also compared by taking into account the surface area. The tables below (Table 2 and Table 3) present the surface area of forest units in consecutive classes of height differences.

In terms of classification based on forest site type, forest units in classes from −1 m to 1 m, i.e., within the range of measurement error, had the largest share (78%) in the analysed area. The largest surfaces in these classes were occupied by the following forest sites: FMCF (295.24 ha) and FPF (528.44 ha). Forest units in positive classes above 1 accounted for 19% of the total analysed area. These were mainly forest sites of the FMHF and FMCF types. On the other hand, forest units in negative classes lower than 1 had a 13% share of the total analysed area. This group included FMHF, MMF, and FMCF sites, but also TASF and AAF types. It is worth noting that in FMHF and FMCF sites, the differences were distributed symmetrically across the whole range. On the other hand, for TASF and AAF sites, the surface area with negative differences was decidedly larger (or even the whole surface for AAF).

As far as the dominant species is concerned, pine occupied the largest surface area in each class. Areas with differences of an absolute value exceeding 2 had only a small share in the whole analysed area. In the classes with positive differences, other species accounted for only a small percentage. A difference of 5 m was noted for spruce in an area of 0.65 ha, which accounts for less than 1% of the total analysed area. A larger variety of species was found in classes of negative differences. Here, differences of an absolute value exceeding 1 m were noted for every species. The total surface area of forest units with negative differences accounted for 13% of the whole analysed area, with pine having the dominant share. Nevertheless, it should be noted that in the majority of forest units (78% of the forest unit area), the differences noted fell into the range of the measurement error, i.e., from −1 m to 1 m.

#### 3.1.2. Comparison of Average Heights for Current Data

The data for the year 2022 were analysed in the same way as the historic data from 2013, i.e., the height of the dominant species from forestry data was deducted from the height from LiDAR data, rounded up to full meters. The largest negative differences reached 8 m, while positive ones reached up to 24 m. Positive values ranging from 8 to 24 m were identified as errors. Similarly, as for the data from 2013, the group with the largest negative errors included fertile and water-related habitats. The errors were assessed by taking into consideration the number of identified crowns in the forest unit (3–177), the age of the forest stand according to the SFIS database (2–15 years), and visual assessment of the tree top point layer on the background of the layer of contours of forest units and non-exclusive areas (NEAs).

The following errors were found that resulted in the misestimation of the height of forest stands:

- Incorrect positioning of the NEA (cluster) on the map; as a result, the height of trees left in the cluster was assumed instead of the height of the forest stand;
- Incorrect contours of the subarea;
- The presence of residual trees, i.e., single trees from an older forest stand, not constituting a cluster (NEA);
- The complex structure of the forest stand, i.e., the presence of sites of additional species, higher than the main forest stand.

**Figure 12:**
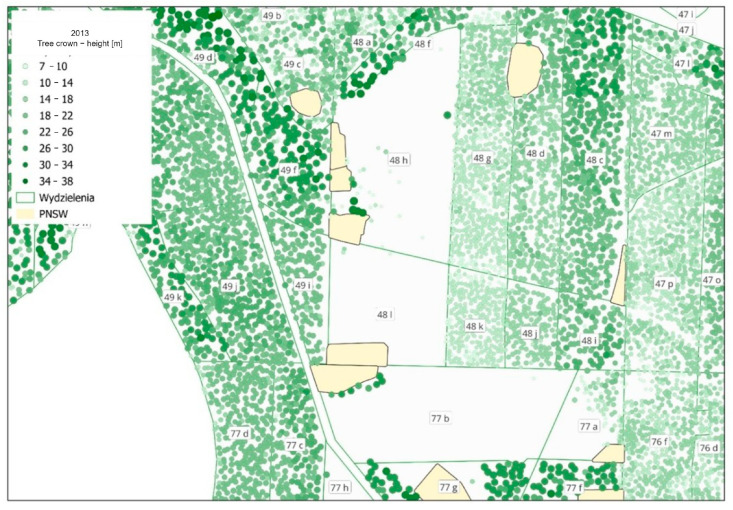
Example of incorrectly estimated heights, resulting from incorrect position of non-exclusive area (48 h, 77 b), borders of forest unit (48 h), and multi-tier forest stand (77 a).

Figure 12 shows examples of errors in height estimation for data from the year 2022.

The height of tree crowns is marked in different shades of green. The incorrect position of the NEA, which is not the same as the actual location of the cluster, is clearly noticeable in forest units 48 h and 77 b. The border between units 48 h and 48 f is also drawn incorrectly, as it is not aligned with the actual border between forest stands. Furthermore, unit 77 a is a forest unit with a multi-tier forest stand, which also makes it impossible to correctly determine the average differences in height for this unit. Sites where the heights were wrongly estimated were excluded from further analyses.

**Figure 13:**
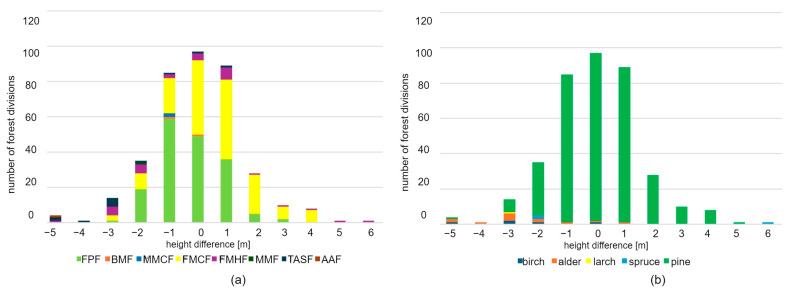
Number of forest units in height difference classes for data from 2022. Forest stand classification based on (**a**) forest site type and (**b**) dominant species.

The number of forest units was analysed for two forest unit classifications: the type of forest site and the dominant species. The results are presented in the diagrams below (Figure 13) and in Table 4 and Table 5. The number of forest units is presented for 14 classes of differences in height. The first class refers to height differences from −8 m to −6 m, while the subsequent classes designate height differences from −6 to 6 m, with an interval of 1 m.

A total of 227 forest units with a total area of 719.09 ha met the required measurement accuracy, which accounts for 65% of the analysed area. Negative values from −2 m to −8 m were noted for 37 forest units with a total area of 94.89 ha, i.e., 8% of the analysed area. Positive values ranging from 2 m to 6 m were noted for 110 forest units (271.19 ha—24% of the analysed area).

The largest negative error (from −6 m to −8 m) occurred in 12 forest units: FMHF—3 forest units (accounting for 12% of all FMHF stands), TASF—8 forest units (57% of all TASF stands), and AAF—1 forest unit (100% of all AAF stands). As for the classification based on dominant species, the largest error was noted for the following forest stands: birch—3 out of 6 forest units, alder—7 out of 14 forest units, spruce—1 out of 3 forest units, and pine—1 out of 351 forest units. The numbers represent, respectively, 60%, 50%, 33%, and less than 1% of all forest units of the same type. The differences take a Gaussian distribution. The most frequent difference in height equals 1 m for both types of classification. The average difference in the height of forest stands is 0.44 m and 0.49 m, respectively, for classification based on forest site type and dominant species. The above results reveal a slight shift towards positive values. However, it does not exceed 1 m, so it falls into the range of an acceptable error. This shift is caused by the maximum number of 1 m differences for the FMCF site type.

The standard deviation was 2.54 m for classification based on habitat type, and 2.11 m for classification based on dominant species. These values are slightly higher than those for data from the year 2013, which suggests that the growth of forest stands was more diversified. In the year 2013, the differences tended to concentrate around the average, while for 2022, more extreme differences, both positive and negative ones, were noted. For example, in 2013, only four forest stands (one birch, two alder, and one pine forest stand) had a height difference of −5 m, and two others (pine and spruce) had a difference of 5 and 6 m, respectively, while in 2022, four forest stands (pine) had a height difference of 5 m, and 17 other forest stands had height differences ranging from −8 to −5 m (3 birch stands, 8 alder, 1 larch, 1 spruce, and 4 pine stands). As far as dominant species are concerned, the group with the largest negative errors included birch and alder. In the year 2022, the LiDAR scan was carried out at the end of April, which is the period when these two species develop leaves and bloom. The absence of a fully leafy crown may have caused the underestimation of height of the extracted tree tops.

Table 4 and Table 5 present the surface areas of forest units in height difference classes for data from the year 2022. The absolute values of height differences for more than half of the forest units (65.4%) did not exceed 1 m. Here, FMCF and FPF stands occupied the largest area, and the classification based on dominant species revealed that pine forest stands represented the majority (691.0 out of 692.1 ha). Forest units in positive classes above 1 accounted for 25.6% of the total analysed area. These were mainly forest sites of FPF and FMCF types. On the other hand, forest units in negative classes lower than −1 m accounted for 8.9% of the total analysed area, with more than half being FPF units with a difference of −2 m. The other half were mainly FMHF units, with differences ranging from −8 m to −5 m, and differences found in every class (however, differences in the class of −8 m to −6 m occupied the largest area (11.1 ha)). As for TASF and AAF, negative differences of an absolute value exceeding −1 m were noted for the whole area.

Both the classification based on type of forest site and that based on dominant species revealed that forest units in the classes from -1 m to 1 m accounted for 65.4% of the whole analysed area. The largest areas in these classes were occupied by the following forest sites: FMCF (244.02 ha) and FPF (427.24 ha). Forest units in positive classes above 1 accounted for 25.6% of the total analysed area, with a large share occupied by FPF and FMCF types. The dominant species was pine. Other species (spruce and larch) occupied only 1.22 ha. On the other hand, forest units in negative classes below −1 accounted for 8.9% of the total analysed surface, with significantly more diversified types of habitats; apart from coniferous species, alder (18.31 ha) and birch (7.14 ha) were also found here. The majority of height differences in coniferous forest stands were positive, while deciduous forest stands had only negative differences in height.

As far as dominant species in forest stands are concerned (considering that this attribute is correlated with the type of forest site), the presence of differences in height was correlated with the dominance of deciduous species. One should also take into consideration the time when the LiDAR scans were performed, March and November, when deciduous species are devoid of their assimilation apparatus. As a result, the laser beam is reflected from the inside of the crown (the trunk and branches) instead of the top layer.

### 3.2. Analysis of Increases in Height

The increases in the height of forest stands in forest units were calculated based on the data from forestry documentation and LiDAR data. The comparison of these increases (Table 6) points to a trend where the values obtained from scanning tend to be higher than those collected from surveys and the SFIS database. The comparison reveals the presence of negative LiDAR values for the growth of 1 m according to forestry data.

For all forest stands in the sample for which a negative or zero increase was recorded in the SFIS database (38 forest units), the LiDAR data from 2013 indicated lower (32 forest units) or equal (6 units) values in comparison to the data from forestry documentation. Surveying works were carried out between the years in which LiDAR scans were taken. These works may have led to the adjustment of previously overestimated heights or may have taken into account the changes that occurred in forest stands (e.g., the dying of spruces). Additionally, this group contains forest stands aged over 100 years (100–205 years old—14 forest units).

The analysis of increases in the height of forest stands based only on LiDAR data demonstrates that negative increases point to a strong correlation with the forest site type and the dominant species. Negative increases were noted for all forest stands in the typical alder swamp forest (TASF) and ash alder forest (AAF) sites, two out of nineteen stands in the fresh mixed hardwood forest (FMHF) site, and one out of two in the moist mixed coniferous forest (MMCF) site. As for the dominant species, it was alder in five cases, birch in two cases, and pine in two cases.

**Figure 14:**
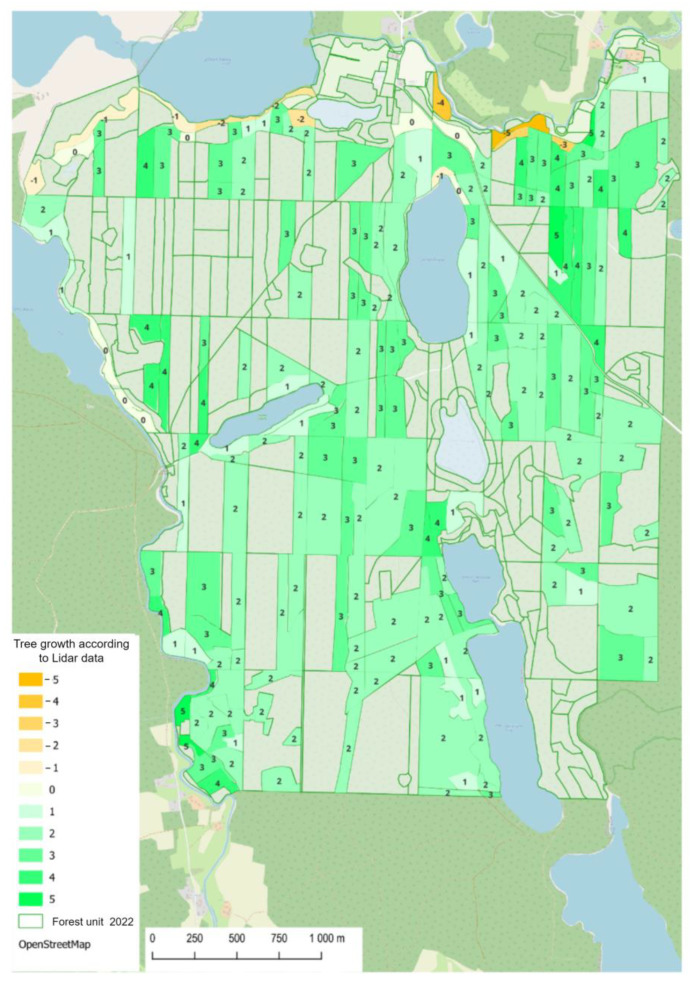
Forest units selected for comparison of height increases with values of height increases based on LiDAR data.

The data on growth increase based on LiDAR were merged with the layer of the selected forest units (Figure 14) and a spatial analysis was performed. Then, the records of the history of forest units and their full composition of species in the SFIS were inspected. Topological analysis revealed that negative increases in height may be divided into two groups: forest units situated along the river valley and those located on a slope overlooking the lake. The same areas also contained zero-growth forest units.

All forest stands situated in the valley of the river are characterised by a very complex composition of species, with a significant dominance of deciduous species (alder and birch). In recent years, these forest stands were exposed to previously unseen fluctuations in the levels of ground and surface waters that resulted from the observed changes in the climate of the region. In the study area, soils are generally poor, sandy, class IV–VI, which can lead to low water retention and frequent soil moisture deficits [48,49,50]. The oldest trees in these forest units are dying, and (due to restrictions on forestry work in water-related habitats) they are not being removed so that they remain there until natural decomposition. On the other hand, the decrease in the height of forest stands on the lake shore results from the damages caused by strong winds that occurred in the years 2017 and 2019.

**Figure 15:**
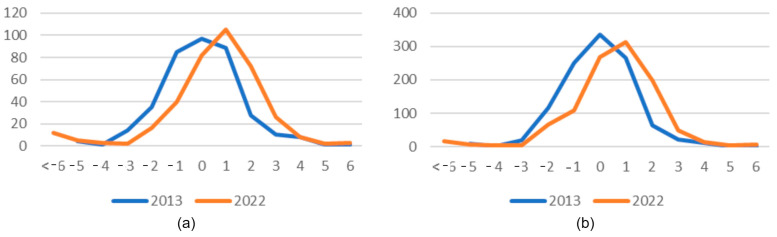
A comparison of the distribution of (**a**) the number and (**b**) the surface area of forest units in height difference classes between the data from forestry documentation and LiDAR data in the years 2013 and 2022.

The diagrams below (Figure 15) present the comparison of the distribution of the number and surface area of forest units in height difference classes in the analysed years (2013 and 2022). The comparison of the quantitative and surface-based distribution of the presence of forest units in forest stand height difference cases based on SFIS data and those determined from LiDAR data in the analysed years reveals a noticeable shift in the curves drawn. SFIS data for the year 2013 are more convergent with LiDAR data. The distribution functions for the year 2013 reach their maximum in class 0. The maximum number of occurrences, both in terms of the number of forest units and their areas, falls into the range with no differences.

For the data from 2022, these maximum values fall into the range of a positive error of 1 m. The curves for the data from the year 2013 have a normal distribution with a maximum in the middle of the range.

Assessment of the conformity of height increments determined using ALS and inventory data was carried out based on Pearson’s correlation coefficient (Equation (10)). The correlation coefficient is a measure of the strength and direction of the linear relationship between two variables. The value of the coefficient ranges from −1 to +1. A value close to +1 indicates a strong positive correlation (an increase in one variable is associated with an increase in the other), a value close to −1 indicates a strong negative correlation (an increase in one variable is associated with a decrease in the other), and a value close to 0 indicates that there is no linear correlation.

(10) $$r_{xy}=\frac{\sum_{i=1}^{n}\left(x_{i}-\bar{x})(y_{i}-\bar{y}\right)}{\sqrt{\sum_{i=1}^{n}{\left(x_{i}-\bar{x}\right)}^{2}\;\sum_{i=1}^{n}{\left(y_{i}-\bar{y}\right)}^{2}}}$$

where n is the number of observations (forest units); $x_{i}$ and $y_{i}$ are height increments in the forest unit determined using ALS data and inventory data, respectively; $\bar{x}$ and $\bar{y}$ are average height increments determined using ALS data and inventory data, respectively. In our experiment, the correlation coefficient between height increments determined for different types of data was 0.6, which is considered a moderate correlation. As shown in Table 6, there were cases (especially with negative increments) where the differences in increments significantly exceeded 1 m. For example, there was one case where the increase according to LiDAR was −5 m, and according to inventory data it was 1 m. Such outliers lowered the value of the Pearson correlation coefficient. For comparison, the correlation coefficient was calculated only for cases where the differences in increments differed by ±1 m, and then it was 0.8. This shows how important the accuracy of LiDAR measurements and the adaptation of the conditions of their acquisition (growing season) to the conditions of inventory data acquisition are for data consistency. In addition, knowledge of any valuation work and changes is crucial for proper evaluation of the results.

Additionally, we calculated other measures of agreement between height increments from LiDAR data and inventory data (such as bias, RMSE, R^2^, rBias) [51]. The bias was 0.2, indicating a slight, systematic overestimation of LIDAR measurements relative to SFIS data. The RMSE was 0.7 m, showing that the average error between stand height increments is less than 1 m. R^2^ was 0.5, which is a moderate value. There is some consistency between the data, but there are also other factors affecting the data. Similarly, an rBias of 12.3% shows moderate consistency and the influence of additional factors. Here, it should be noted that both LiDAR and inventory data are affected by uncertainty. Inventory errors and the relatively low resolution of ALS data may further underestimate the consistency between the determined increments (especially in the case of metrics based on deviations). Nevertheless, the correlation coefficient and R^2^ confirm moderate agreement between the determined tree height increments, and the high accuracy of LiDAR data can significantly improve these results.

### 3.3. Analysis of Forest Site Conditions of the Forest Units

The height of forest stands of a given age is one of the main attributes that provide information about the potential of the forest site in a given location. The obtained point data on the height of crowns in forest units enable us to analyse the variability in the forest site within its borders and therefore to improve the planning of management activities. This applies particularly to the renewal of forests via partial cutting (with the aim of transforming uniform pine stands into forest stands of a complex age structure, spatial structure, and composition of species), as the places where sites are established (that are cut first and renewed with deciduous species) should be located in areas that offer the best conditions for growth for the young generations of the forest.

**Figure 16:**
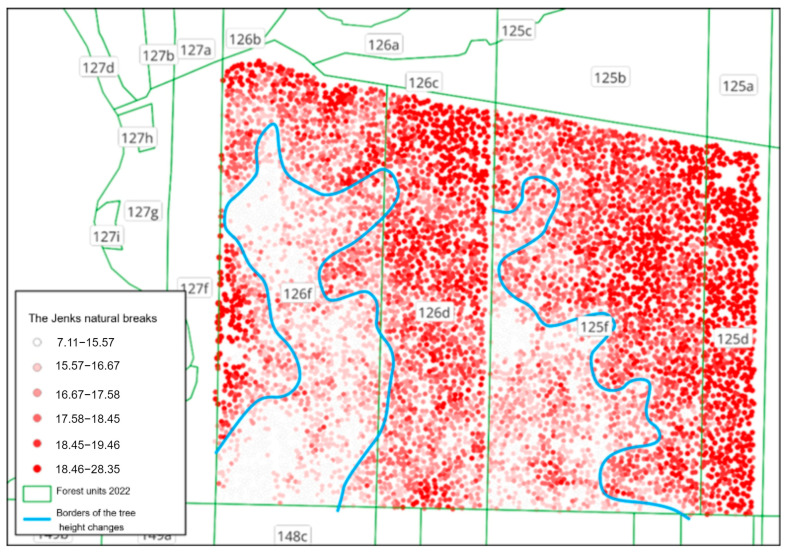
Analysis of local variability in site potential with the use of Jenks natural breaks method.

The variability in habitat properties is visualised in Figure 16 using Jenks natural breaks. This is a method of data classification that minimises intra-group variance and maximises inter-group variance. This method enables us to distinguish the areas with similar habitat properties (areas with low intra-group variance) and, at the same time, to determine the borders between areas of different habitat conditions (high variance between neighbouring groups of properties). The observation of changes in various ranges of values enables to note the tendency of clustering values and thus to determine the areas that differ in terms of site potential.

The Jenks natural breaks method applied here allows us to search for clusters of values in a group, so as to distinguish data categories. In the analysed case, areas with lower trees may be observed within forest units 125 f and 126 f (i.e., theoretically, within an area of uniform forest stand). The difference in height enables us to draw conclusions concerning the potential of the site (trophic, water-related, maybe connected with terrain formation—e.g., the top of a dune, from which organic elements of soil are washed down). This type of analysis may provide guidelines for further cultivation activities in the forest and/or for the selection of the distribution of species during the process of renewal after felling or transforming the forest stand. It should be noted, however, that the Jenks natural breaks method is exploratory in nature, as it is used to identify natural clusters of values in the data without making any prior statistical assumptions, thus allowing for the recognition of the internal structure of habitat variability. Therefore, the Jenks method serves only as a tool for discovering potential differences in habitat potential, which may later form the basis for further, more detailed studies.

### 3.4. Assessment of Tree Top Detection Accuracy

In our experiment, we calculated the DTM, DSM, and CHM with a resolution of 0.5 m. For comparison, we also generated the CHM with a resolution of 1 m and examined the accuracy of these models. We selected 20 test areas with different stand properties (different species, different tree ages, different heights). The heights read from the model were compared with field data. The CHM with a pixel size of 0.5 m achieved a vertical accuracy of 1.5 m, while the CHM with a pixel size of 1 m achieved an accuracy of 1.2 m, with a standard deviation of 1.3 m and 1.2 m, respectively. These results show that the accuracy of both canopy models is similar. The lower resolution of the model (1 m) allows for greater generalization, which results in the elimination of some outliers, which in turn gives a slightly higher accuracy of the model along the Z coordinate.

In addition, we examined the precision and sensitivity of the tree top detection methodology used. We selected 15 test areas for which we manually digitized tree tops based on UAV and LiDAR data. These tops were compared with the results of automatic detection, and then we calculated the metrics listed in Table 7. Precision determines how many of the predicted positive examples are actually positive. The maximum value (1) indicates complete agreement between the predicted and reference data. Recall measures the number of actual positive examples that the model detected correctly. The F1-score is the harmonic mean of precision and sensitivity. It is particularly useful for imbalanced data. Root mean square error (RMSE) measures the error between reference data and actual data; its value depends on both the prediction methodology used and the accuracy of the reference data obtained.

Precision in both cases was 0.8, and the F1-score was 0.7. These are high values and indicate a high degree of consistency between the detected vertices and reality. A lower value was achieved for Recall, which shows that not all actual tree crowns were automatically digitized. The RMSE was also around 12 and 10 trees (for 2013 and 2022, respectively), with these results being influenced by a large number of undetected tree tops in areas of young, dense forest.

The results show that there were relatively few false detections. The peaks detected by the algorithm were largely consistent with reality. On the other hand, however, there were quite a few reference points without a match, which was due to two reasons. First, the reference data were subject to observer errors, and some spreading deciduous trees in the UAV images could have been detected as several vertices. Second, the ALS data had a resolution of 4 points/m^2^, which did not allow for the detection of trees with a small surface area (hence the larger errors for young forests).

In addition, we checked the uncertainty of inventory data in our experiment. This uncertainty was estimated assuming an error of σ = 0.3 m per tree. The average stand height $\overset{¯}{H}$ is the weighted average of the heights of the dominant species and their share in the forest stand.

(11) $$\overset{¯}{H}=\frac{\sum n_{i}h_{i}}{\sum n},$$

where $n_{i}$ is the number of trees with a height $h_{i}$; n is the total number of trees in the stand. The uncertainty of this mean (SE) is as follows:

(12) $$SE=\;\frac{\sigma }{\sqrt{n}}$$

Next, the uncertainty of determining the height of the tree stand using the Monte Carlo method (1000 random samples) was propagated. The analysis showed that the uncertainty of the average height of the tree stand is negligible (<0.02 m), because the number of trees is very large (on average, about 1000 trees in the tree stand). The Monte Carlo method confirms that the distribution of averages is close to normal and very narrow.

## 4. Discussion

The analysis of consistency between field inventory data and airborne laser scanning (ALS/LiDAR) data in the context of assessing tree height and growth is an important and practical area of research in forestry. Rahman et al. [52] tested whether ALS is useful for determining tree heights in Japan. Similar studies for Poland were conducted by P. Hawryło et al. [53]; however, they compared the heights of forest stands, with individual heights determined using LiDAR. For the maximum height of automatically detected tree tops, they achieved an RMSE of 0.29 m. We compare the height increments of forest stands determined using LiDAR, with the height increments of the same forest stands determined during field inventory (collected in forest documentation).

We applied an innovative approach to assess the growth of various types of forest stands based on data from airborne laser scanning. The research was conducted based on LiDAR point clouds with an average density of 4 pts/m^2^ and an accuracy of 0.15 m–0.25 m. The LiDAR data charts downloaded from the national geoportal were subjected to the following operations: classification, rasterization, and DTM or DSM generation, followed by their subtraction (CHM = DSM − DTM). After that, a Gaussian smoothening filter was applied, the trees below the value of 6 m were rejected, and finally the data were exported to a text file. The calculated values of forest stand heights were compared to reference data obtained from forestry documentation.

The most frequent height differences (between the ALS and inventory data) equal to 0 m (for 2013) and 1 m (for 2022) show a high degree of consistency between the heights determined from the compared data. The average values for both dates do not exceed 1 m, so they are within the acceptable error limit. The average values close to 0 m show that there was no systematic shift between the data types compared, which also confirms their agreement. Higher standard deviations for 2022 (approximately 2 m), on the other hand, show a greater spread of data around the mean value. In 2022, there were more differences with extreme values, both positive and negative. This is probably due to errors in the reflection of the laser beam. A correlation was observed between the appearance of greater height differences and the prevalence of deciduous species. The ALS data for 2022 was obtained at the end of April during the development of leaves and flowering of these species. Our research has shown that tree species significantly affect the accuracy of measurement. Ivanovs et al. [54] showed that the species composition of the forest significantly affects the accuracy of height determination from ALS data. The RMSE ranged from 1.4 m for pine stands during the growing season to 3.8 m for birches during the leafless season.

We also calculated Pearson’s correlation coefficient between forest stand height increments determined using ALS data and inventory data to verify the consistency of these data. Pearson’s correlation coefficient between these data was 0.6, which is considered a moderate correlation. Relative studies comparing inventory data and ALS in the context of tree height and growth measurements show moderate agreement, with root mean square errors (RMSEs) for height and weaker correlations for growth measurements [55].

Guerra-Hernández and Pascual [56] showed in their research that the median growth rates calculated from LiDAR and inventory data were similar. Montzka et al. [57] also showed that the average tree height did not differ significantly between ALS and inventory data. This shows that it is possible to improve forest inventory using ALS data. However, the correlation is not strong enough to consider completely replacing field measurements. More research will likely determine to what extent these methods could replace each other. Performing LiDAR measurements during the growing season could minimize errors in determining tree height and increase the correlation between ALS and inventory data.

Authors of publications on similar topics point out the following [54,55]:

- The use of ALS for tree height measurements due to its higher accuracy and reliability.
- A cautious approach to growth measurements, especially over short periods.
- Taking into account species composition and stand conditions when choosing a growth assessment method.

The application of LiDAR data will improve the adequacy of survey descriptions. Therefore, planning tasks in forest management will reflect the needs and production capacity of forest stands to a more satisfying degree. LiDAR data may constitute the basis for updating the SFIS database with respect to survey descriptions early in the course of executing the Forest Management Plan. The possibility of identifying local variations in forest sites will contribute to improving their potential, which, in turn, will translate to improved resistance of forest stands to environmental conditions. LiDAR data collected in different years offer an insight into the trends in the changes that occur in forest stands. This refers both to changes caused by climate conditions and those that result from the ongoing forest management. Apart from the possibility of identifying the form of the top layer of crowns, LiDAR data also offer information about the inside of the forest stand. The possibility of detecting tree trunks based on a projection of a section of the point cloud from ground level to the beginning of the crown would allow for more precise detection of crown tops and determination of the number of trees. Furthermore, developing algorithms that would link the biosocial class of the tree to the resulting potential crown size might enable precise division of the crown layer into individual trees in the form of vector polygons and allow us to assign attributes to them based on multi-spectral imaging. Due to the fact that they enable us to identify tree species based on the characteristics of reflection in specific ranges, it would become possible to prepare a survey description for the upper layer of the forest stand, with respect to the composition of species, the share of individual species, and the degree of afforestation and density of the forest stand. Automation of some of the surveying works would reduce the costs of preparing the Forest Management Plan, while the availability of remote sensing data would enable us to improve the control of the parameters that influence methods of forest management.

Although LiDAR technology offers significant advantages as far as tree height measurement is concerned, some challenges still exist, in particular in complex forest habitats, where the diversity of species and terrain may make data interpretation more difficult. Similar conclusions were drawn by the authors of [58]. In order to reduce the measurement error in assessing tree height with the use of LiDAR technology, LiDAR data may be integrated with high-resolution images.

## 5. Conclusions

The aim of this study was to analyse the increase in the height of forest stands based on LiDAR data and to compare the results with the growth calculated based on inventory data collected by the State Forest Information System (in Poland). The height of the tree, along with the diameter at breast height (DBH), is a key parameter that influences the calculated volume and yield of the forest stand. Traditional methods of measuring tree height may carry a risk of errors, which, in turn, may lead to the misestimation of wood yield and thus to incorrect planning of forest management. The study was based on publicly available data obtained from laser scanning at aviation altitude in order to compare data from direct measurement with LiDAR data. The research was conducted in the Przymuszewo Forest District, in Zbrzyca forest range. The obtained results revealed that LiDAR data may be applied, after processing with the use of open and free software, to determine the height of forest stands and its increase, and therefore to automate some of the survey works. This may contribute to reducing the costs of forest surveying and improving the control over the parameters that influence forest management.

In summary, LiDAR data are a valuable source of information about the vertical structure of tree stands and enables precise determination of their height, but their use has certain limitations. The accuracy of the results depends primarily on the quality of the data, the density of the point cloud, and the processing methods used. Measurement errors are also influenced by stand characteristics such as species diversity, density, and the presence of undergrowth, as well as environmental conditions at the time of measurement.

Despite these limitations, LiDAR technology remains an extremely useful tool in forest research, especially when combined with field data that allow for the validation and calibration of height models, increasing the reliability of the results obtained.

Future research on the use of ALS (airborne laser scanning) data to determine stand growth should focus on increasing the frequency of measurements and standardizing data processing methods, which will allow for more precise comparisons of results over time. Integrating ALS data with other sources, such as ground-based and drone-based LiDAR (TLS, UAV-LiDAR), is also an important direction. Further research should also focus on improving tree segmentation and analysis algorithms using artificial intelligence and machine learning methods, which can significantly improve the accuracy of tree growth and increment modelling. Equally important is research on the calibration and validation of LiDAR data using field measurements and analysing the impact of environmental factors such as soil conditions, moisture, and climate change. Combining these approaches could contribute to the development of more reliable and universal stand growth models in the future, supporting sustainable forest management.

## Figures and Tables

**Table 1:** Classes of LiDAR points.

Class	Types of Classified Objects
0	Points processed, but not classified
2	Points situated on the ground
3	Points that represent low vegetation, i.e., from 0.0 to 0.40 m
4	Points that represent medium vegetation, i.e., from 0.40 to 2.00 m
5	points that represent tall vegetation, i.e., above 2.00 m
6	Points that represent buildings, structures, and civil engineering objects
7	Noise
8	Points that represent areas covered with water
12	Points from multiple overlap areas

**Table 2:** Surface of forest units [ha] in height difference classes, classified based on forest site type—2013: fresh mixed hardwood forest (FMHF), fresh mixed coniferous forest (FMCF), fresh pine forest (FPF), moist mixed coniferous forest (MMCF), boggy mixed forest (BMF), typical alder swamp forest (TASF), mixed moist forest (MMF), and ash alder forest (AAF).

Forest Site Type	Difference in Height: Inventory Data—LiDAR [m]											
	−5	−4	−3	−2	−1	0	1	2	3	4	5	6
	Surface Area of Forest Units [ha]											
FPF			1.45	85.3	194.08	204.45	129.91	9.26	1.74			
BMF					0.55	0.92						
MMCF					1.94							
FMCF			2.59	22.23	49.46	122.27	123.51	52.87	17.72	10.04		
FMHF	1.12		6.18	5.93	2.33	5.79	11.61	1.16	1.22	0.8	0.26	0.65
MMF				0.54								
TASF	5.19	1.03	7.54	1.8	1.69	2.34	1.86					
AAF	1.21											
TOTAL	7.52	1.03	17.76	115.8	250.05	335.77	266.89	63.29	20.68	10.84	0.26	0.65

**Table 3:** Surface area [ha] of forest units in height difference classes based on dominant species—2013.

Dominant Species	Difference in Height: Inventory Data—LiDAR [m]											
	−5	−4	−3	−2	−1	0	1	2	3	4	5	6
	Surface Area of Forest Units [ha]											
Birch	3.66		2.23	1.16		1.05						
Alder	2.74	1.03	5.94	2.34	1.69	2.34	1.86					
Larch			0.99									
Spruce			2.96	2.36								0.65
Pine	1.12		8.6	109.34	248.36	332.38	265.03	63.29	20.68	10.84	0.26	
TOTAL	7.52	1.03	17.76	115.8	250.05	335.77	266.69	63.29	20.68	10.84	0.26	0.65

**Table 4:** Surface of forest units [ha] in height difference classes, classified based on forest site type for 2022: fresh mixed hardwood forest (FMHF), fresh mixed coniferous forest (FMCF), fresh pine forest (FPF), moist mixed coniferous forest (MMCF), boggy mixed forest (BMF), typical alder swamp forest (TASF), mixed moist forest (MMF), and ash alder forest (AAF).

Forest Site Type	Difference in Height: Inventory Data—LiDAR [m]												
	−8 to −6	−5	−4	−3	−2	−1	0	1	2	3	4	5	6
	Surface Area of Forest Units [ha]												
FPF			0.45		51.91	61.87	186.03	179.34	80.04	18.54		1.76	2.91
BMF						0.55	0.99						
MMCF		0.77			0.76			1.23					
FMCF					8.67	40.59	72.51	130.92	116.6	28.25	12.93	0.74	3.87
FMHF	3.81	3.11			1.82	5.08	9.91	3.07	2.81	1.94	0.8		
MMF				0.54									
TASF	11.08	3.42	2.83	2.34	2.19								
AAF	1.21												
TOTAL	16.1	7.3	3.28	2.88	65.35	108.09	269.44	314.56	199.45	48.73	13.73	2.5	6.87

**Table 5:** Surface area [ha] of forest units in height difference classes based on dominant species—2022.

Dominant Species	Difference in Height: Inventory Data—LiDAR [m]												
	−8 to −6	−5	−4	−3	−2	−1	0	1	2	3	4	5	6
	Surface Area of Forest Units [ha]												
Birch	5.92				1.22		1.05						
Alder	6.99	3.42	2.83	2.88	2.19								
Larch		0.99									0.5		
Spruce	2.36				0.6					0.72			
Pine	0.83	2.89	0.45		61.34	108.09	268.39	314.56	199.45	48.01	13.23	2.5	6.87
TOTAL	16.1	7.3	3.28	2.88	65.35	108.09	269.44	314.56	199.45	48.73	13.73	2.5	6.87

**Table 6:** Number of forest units with height increases.

Number of Forest Units		Increase in Height Based on LiDAR Data										
		−5	−4	−3	−2	−1	0	1	2	3	4	5
Increase in height based on forestry documentation	−1				1			1				
	0				1	2	4	8	20	1		
	1	1	1	2	1	2	1	9	35	6		
	2						3	7	31	29	6	
	3								2	18	11	3
	4								2	5	2	
	8											1

**Table 7:** Sensitivity and accuracy metrics for tree top detection for 2013 and 2022 datasets.

	2013	2022
Precision	0.8	0.8
Recall	0.6	0.7
F1_score	0.7	0.7
RMSE	11.9	9.5

## Data Availability

The original contributions presented in this study are included in the article. Further inquiries can be directed to the corresponding author.

## Abbreviations

The following abbreviations are used in this manuscript:

LiDAR	Light Detection and Ranging
UAV	Unmanned Aerial Vehicle
RGB	Red–Green–Blue
NIR	Near Infrared
GEDI	Global Ecosystem Dynamics Investigation
ALS	Aerial Laser Scanning
ULS	Unmanned Laser Scanning
SFIS	State Forest Information System
NEA	Non-Exclusive Area
DRFC	Data for Reports on Forest Condition
ASPRS	American Society for Photogrammetry and Remote Sensing
GPS	Global Positioning System
FDM	Forest Digital Map
WMS	Web Map Service
WFS	Web Feature Service
DSM	Digital Surface Model
DTM	Digital Terrain Model
TIN	Triangulated Irregular Network
CHM	Canopy Height Model
FMHF	Fresh Mixed Hardwood Forest
FMCF	Fresh Mixed Coniferous Forest
FPF	Fresh Pine Forest
MMCF	Moist Mixed Coniferous Forest
BMF	Boggy Mixed Forest
TASF	Typical Alder Swamp Forest
MMF	Mixed Moist Forest
AAF	Ash Alder Forest
